# MicroRNA deregulation in triple negative breast cancer reveals a role of miR-498 in regulating *BRCA1* expression

**DOI:** 10.18632/oncotarget.7705

**Published:** 2016-02-25

**Authors:** Nerea Matamala, Maria Teresa Vargas, Ricardo González-Cámpora, Jose Ignacio Arias, Primitiva Menéndez, Eduardo Andrés-León, Kira Yanowsky, Ana Llaneza-Folgueras, Rebeca Miñambres, Beatriz Martínez-Delgado, Javier Benítez

**Affiliations:** ^1^ Human Cancer Genetics Programme, Spanish National Cancer Research Centre (CNIO), Madrid, Spain; ^2^ Pathology Service, Hospital Virgen de la Macarena, Sevilla, Spain; ^3^ Surgery Service, Hospital Monte Naranco, Oviedo, Spain; ^4^ Pathology Service, Hospital Monte Naranco, Oviedo, Spain; ^5^ Bioinformatics Unit, Spanish National Cancer Research Centre (CNIO), Madrid, Spain; ^6^ General Surgery Service, Hospital Universitario Central de Asturias, Oviedo, España; ^7^ Projects Unit, Sistemas Genómicos, Valencia, Spain; ^8^ Molecular Genetics Unit, Research Institute of Rare Diseases (IIER), Instituto de Salud Carlos III (ISCIII), Madrid, Spain; ^9^ Network on Rare Diseases (CIBERER), Madrid, Spain; ^10^ Current address: Molecular Genetics Unit, Research Institute of Rare Diseases (IIER), Instituto de Salud Carlos III (ISCIII), Madrid, Spain; ^11^ Current address: Hematology Service, Hospital Virgen de la Macarena, Sevilla, Spain; ^12^ Current address: Bioinformatics Unit, Instituto de Parasitología y Biomedicina “López Neyra”, Consejo Superior de Investigaciones Científicas (IPBLN-CSIC), Granada, Spain

**Keywords:** BRCA1, microRNA, triple negative breast cancer

## Abstract

Emerging evidence suggests that BRCA1 pathway contributes to the behavior of sporadic triple negative breast cancer (TNBC), but little is known about the mechanisms underlying this association. Considering the central role that microRNAs (miRNAs) play in gene expression regulation, the aim of this study was to identify miRNAs specifically deregulated in TNBC and investigate their involvement in *BRCA1* regulation. Using locked nucleic acid (LNA)-based microarrays, expression levels of 1919 miRNAs were measured in paraffin-embedded tissues from 122 breast tumors and 11 healthy breast tissue samples. Differential miRNA expression was explored among the main subtypes of breast cancer, and 105 miRNAs were identified as specific for triple negative tumors. *In silico* prediction revealed that miR-498 and miR-187-5p target *BRCA1*, and these results were confirmed by luciferase reporter assay. While miR-187-5p was found overexpressed in a luminal B cell line, miR-498 was highly expressed in a triple negative cell line, Hs578T, and its expression was negatively correlated with the levels of *BRCA1*. We functionally demonstrated that miR-498 inhibits *BRCA1* in breast cancer cell lines, and showed that inhibition of miR-498 led to reduced proliferation in the triple negative cell line Hs578T. Our results indicate that miR-498 regulates *BRCA1* expression in breast cancer and its overexpression could contribute to the pathogenesis of sporadic TNBC via *BRCA1* downregulation.

## INTRODUCTION

Breast cancer is a highly heterogeneous disease, both histological and clinically. Gene expression profiling has demonstrated the existence of at least four major types with distinct biological features, clinical outcomes and responses to therapies: luminal A, luminal B, Her2 and basal-like/triple negative [1, 2]. Among them, TNBC represents a treatment challenge because these cancers do not benefit from hormonal or Her2-targeted therapies. Moreover, they have a more aggressive clinical behavior and are associated with a higher recurrence rate and shorter survival [3]. *BRCA1* gene is a tumor-suppressor gene frequently mutated in hereditary breast cancer. BRCA1 plays a critical role in various cellular processes, including among others DNA repair by distinct pathways, cell cycle checkpoints control, centrosome amplification, transcriptional activation or ubiquitin ligation [4, 5]. Sporadic triple negative tumors share many characteristics with *BRCA1*-germline mutated breast tumors [6] but they usually do not present somatic mutations in the *BRCA1* gene. However, a reduction in *BRCA1* expression has been shown [7-9], which suggests a possible role of BRCA1 dysfunction in the pathogenesis of sporadic TNBC. This low expression has been associated with promoter hypermethylation, loss of heterozygosity (LOH) and overexpression of two proteins: HMGA1 and ID4 [7, 8]. Nevertheless, other mechanisms such as miRNA deregulation might also be involved in the inactivation of *BRCA1* in sporadic triple negative tumors [10].

In this way several studies have identified aberrantly expressed miRNAs in breast tumors when compared with healthy breast tissues [11-17], but little is known about differential miRNA expression between breast cancer molecular subtypes [18]. Characterization of subtype-linked miRNAs is of relevance since they might provide better understanding of the biology of these groups of tumors. This is especially important in the case of triple negative cancers, which do not respond to current targeted therapies, and thus identification of deregulated miRNAs could have important therapeutic implications.

In the present study, we aimed to identify miRNAs specifically deregulated in TNBC and investigated their involvement in *BRCA1* regulation.

## RESULTS

### miRNAs specifically deregulated in TNBC

**Figure 1:**
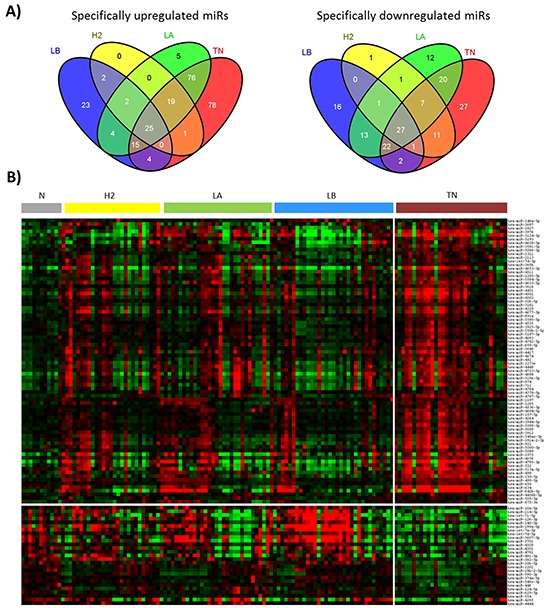
Subtype-specific miRNAs in 122 breast tumors **A.** Venn diagram showing miRNAs specifically up or downregulated in each molecular subtype, and those commonly deregulated in all the subtypes. **B.** Heatmap showing the relative expression of 105 miRNAs specific for triple negative tumors. Red color indicates over-expression and green, repression. TN: triple negative, H2: Her2, LB: luminal B, LA: luminal A, N: normal breast tissues.

We previously have performed miRNA expression profiling of 122 breast tumors and 11 healthy breast tissues and have identified miRNAs associated with breast cancer [17]. Now, to further characterize the miRNA expression profile of breast tumors, we have compared each subtype with the healthy breast tissues and identified 335, 98, 157 and 249 differentially expressed (FDR<0.05) miRNAs in triple negative, Her2, luminal B and luminal A tumors, respectively. We analyzed these results by using a Venn diagram and obtained 105, 1, 39 and 17 miRNAs (up and downregulated) specific for triple negative, Her2, luminal B and luminal A tumors, respectively (Figure 1A, Supplementary Table S1).

Since we were interested in miRNAs associated with TNBC, we selected the triple negative-specific miRNAs (Figure 1B) and investigated biological processes that are predicted to be targeted collectively by these miRNAs by using Diana miRPath web-based computational tool. The pathway analysis revealed statistically significant enrichment for 100 pathways related to proliferation, signaling and migration. The top 10 associated functions are shown in Table 1.

**Table 1:** Top 10 significantly enriched signaling pathways associated with the miRNAs specific for triple negative tumors

KEGG pathway	p-value	#genes
PI3K-Akt signaling pathway	2.43E-38	184
MAPK signaling pathway	1.26E-29	137
Regulation of actin cytoskeleton	1.25E-25	116
Endocytosis	3.09E-25	109
Focal adhesion	1.31E-24	116
Calcium signaling pathway	6.45E-23	104
Wnt signaling pathway	1.29E-21	93
RNA transport	3.24E-18	81
Ubiquitin mediated proteolysis	3.24E-18	83
Dopaminergic synapse	1.27E-17	79

### *BRCA1* is a target of miR-498 and miR-187-5p

**Figure 2:**
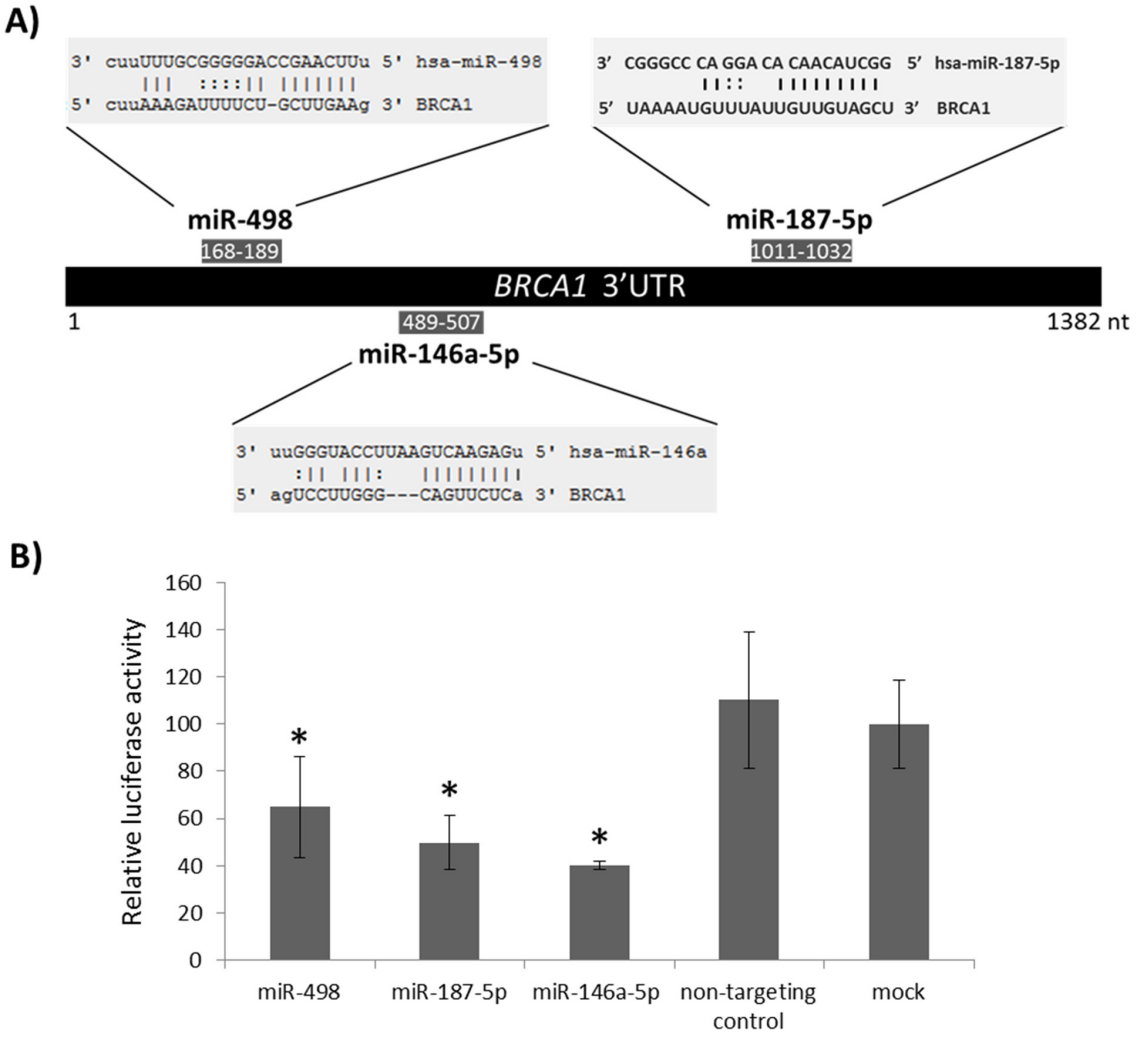
Targeting of *BRCA1* 3′ UTR by miR-498 and miR-187-5p **A.** Schematic representation of miRNA binding sites within the *BRCA1* 3′UTR. **B.** Relative luciferase activity of a reporter vector carrying the *BRCA1* 3′UTR downstream of the firefly luciferase gene. The vector was co-transfected with each of the indicated miRNA precursors or with no miRNA precursor (mock) into 293T cells. Error bars represent standard deviation for four replicates of one representative experiment. Data were normalized versus the luciferase levels generated by the mock transfection. *p<0.05.

Considering that reduced expression of *BRCA1* in sporadic triple negative tumors could be produced by high levels of one or several miRNAs targeting this gene, we investigated if any of the 78 miRNAs specifically upregulated in sporadic triple negative tumors have binding sites in the 3′UTR of *BRCA1*. Five different target prediction algorithms as included in miRGate [19] (Miranda, Pita, TargetScan, Microtar and RNAhybrid) were used. Sixteen miRNAs were predicted to bind to the 3′UTR of the *BRCA1* gene, being miR-498, miR-187-5p and miR-146a-5p the miRNAs with higher scores (Table 2, Figure 2A). Since miR-146a-5p has already been reported to regulate *BRCA1* expression [10], we selected miR-498 and miR-187-5p for further investigation and used miR-146a-5p as a positive control.

**Table 2:** miRNAs specifically overexpressed in triple negative breast tumors that are predicted to target the 3′UTR of *BRCA1*

miRNA	Start	Stop	Target-site	Agreement	Prediction algrithms	Agreement score
hsa-mir-187-5p	1011	1032	7mer-m8	1	Miranda	1,264
hsa-mir-498	168	189	7mer-m8	1	Miranda	1,236
hsa-mir-146a-5p	489	507	8mer	3	Miranda, Mirtarbase, OncomiRDB	1,209
hsa-mir-2113	198	220	8mer	1	Miranda	1,199
hsa-mir-4639-3p	439	456	7mer-m8	1	Miranda	1,195
hsa-mir-4503	167	188	N/A	1	Miranda	1,182
hsa-mir-4653-3p	972	997	N/A	1	Miranda	1,182
hsa-mir-3976	293	319	N/A	1	Miranda	1,113
hsa-mir-2113	332	354	N/A	1	Miranda	1,099
hsa-mir-1285-5p	829	847	Offset 3-8 6mer	1	Miranda	1,099
hsa-mir-921	978	1003	N/A	1	Miranda	1,086
hsa-mir-5000-3p	89	110	7mer-m8	1	Miranda	1,072
hsa-mir-5580-5p	1	12	7mer-m8	1	Miranda	1,058
hsa-mir-150-5p	599	620	7mer-m8	1	Miranda	1,044
hsa-mir-1197	924	944	N/A	1	Miranda	1,044
hsa-mir-498	1058	1080	N/A	1	Miranda	1,044
hsa-mir-146a-5p	983	989	8mer	3	Pita, Mirtarbase, OncomiRDB	−0,868
hsa-mir-3925-5p	984	990	8mer	1	Pita	−0,868
hsa-mir-548ao-3p	982	988	8mer	1	Pita	−0,868

We investigated whether the 3′UTR of *BRCA1* is a functional target of miR-498 and miR-187-5p by using a reporter vector into which the entire 3′UTR of *BRCA1* was inserted downstream of the firefly luciferase reporter gene. This reporter vector was transiently transfected into 293T cells together with pre-miR-498, pre-miR-187-5p, non-targeting control, positive control (pre-miR-146a-5p) or no miRNA precursor. An average of 35% and 50% reduction of reporter activity as compared to the mock transfection control was observed for miR-498 and miR-187-5p, respectively (Figure 2B), indicating that these miRNAs target *BRCA1* 3′UTR. Similar to previous studies [10, 20], the degree of luciferase inhibition with miR-146a-5p reached 60%.

### MiR-498 and miR-187-5p expression in breast tumors and breast cancer cell lines of different subtypes

**Figure 3:**
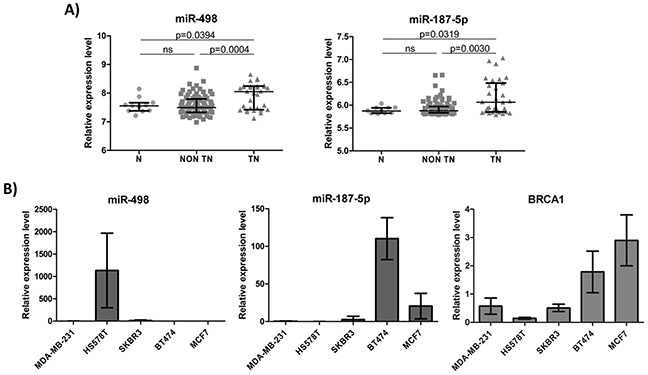
miR-498 and miR-187-5p expression levels in breast tumors and breast cancer cell lines of different subtypes **A.** Relative expression of miR-498 and miR-187-5p in 11 normal breast tissues (N), 91 non triple negative tumors (non TN) and 31 triple negative tumors (TN). **B.** Relative expression of miR-498, miR-187-5p and *BRCA1* in two triple negative (MDA-MB-231 and Hs578T), one Her2 (SKBR3), one luminal B (BT474) and one luminal A (MCF7) breast cancer cell lines. Error bars represent standard deviation for triplicates of one representative experiment.

We next analyzed the expression of miR-498 and miR-187-5p in breast tumors and breast cancer cell lines of different subtypes. As mentioned before, miR-498 and miR-187-5p expression levels were increased in sporadic triple negative breast tumors but not in other subtypes when compared with healthy breast tissues (Figure 3A). Regarding breast cancer cell lines, we found that miR-498 was expressed at high levels in a triple negative cell line (Hs578T) while miR-187-5p was highly expressed in a luminal cell line (BT474). In addition, lower expression levels of *BRCA1* were found in the triple negative and the Her2 cell lines when compared with the luminal cell lines (Figure 3B). Because we were interested in miRNAs with increased expression levels in triple negative cell lines and negatively correlated with *BRCA1* expression levels, we decided to focus on miR-498 for following experiments.

### MiR-498-mediated inhibition of BRCA1 in breast cancer cell lines

**Figure 4:**
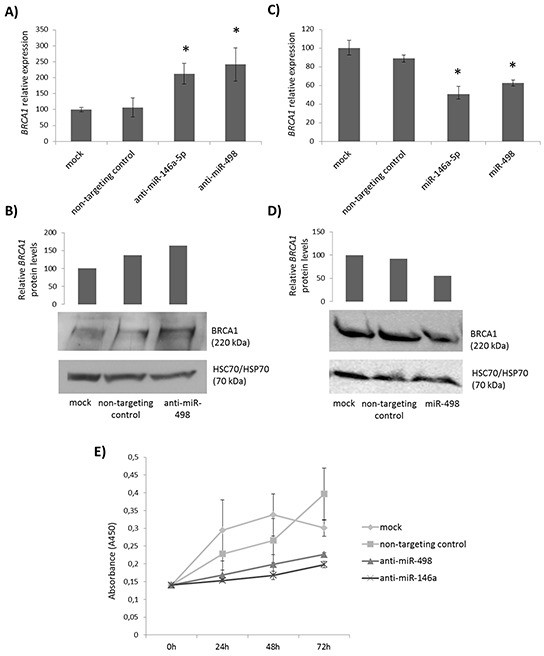
Effect of miR-498 expression and inhibition on *BRCA1* expression and cell proliferation **A.** Relative mRNA levels of *BRCA1* after transfection of Hs578T cells with anti-miR-498, anti-miR-146a-5p, non-targeting control or no miRNA inhibitor. **B.** Western blot analysis of BRCA1 expression in Hs578T cells after transfection with anti-miR-498, non-targeting control or no miRNA inhibitor. Full-length BRCA1 was detected using a monoclonal anti-BRCA1 antibody (Calbiochem, #OP92) and HSC70/HSP70 served as a loading control. **C.** Relative mRNA levels of *BRCA1* after transfection of MCF7 cells with pre-miR-498, pre-miR-146a-5p, non-targeting control or no miRNA precursor. **D.** Western blot analysis of BRCA1 expression in MCF7 cells after transfection with pre-miR-498, non-targeting control or no miRNA precursor. **E.** Effect of miR-498 inhibition on proliferation of Hs578T cells. WST-1 cell viability assay was performed at 24, 48 and 72 hours after transfection of Hs578T cells with anti-miR-498, anti-miR-146a-5p, non-targeting control or no miRNA inhibitor. Error bars represent standard deviation for triplicates of one representative experiment. * p<0.05.

Since miR-498 was expressed at high levels in Hs578T cells and at low levels in MCF7 cells, we next investigated the consequences of miR-498 inhibition in Hs578T cell line and of miR-498 overexpression in MCF7 cell line. We selected MCF7 cell line to overexpress miR-498 since it expressed the highest levels of *BRCA1* and therefore the effect of miR-498 expression on *BRCA1* levels could be clearly observed. As expected, miR-498 inhibition led to an increase in the amount of BRCA1 (141% increase at the mRNA level (Figure 4A) and 64% increase at the protein level (Figure 4B)) while its overexpression produced a reduction of BRCA1 (38% decrease at the mRNA level (Figure 4C) and 44% decrease at the protein level (Figure 4D)), as compared with mock transfection. These results demonstrate that miR-498 inhibits *BRCA1* expression in breast cancer cells.

### Inhibition of miR-498 reduced proliferation in triple negative cells

To gain more insight into the biological effect of miR-498 on breast tumorigenesis and given that BRCA1 is presumed to have a growth suppressor function [21, 22], we transfected Hs578T cells, which previously showed elevated levels of miR-498, with anti-miR-498, anti-miR-146a-5p, non-targeting miRNA inhibitor or mock control, and analyzed cell growth by WST-1 cell viability assay. Figure 4E shows that inhibition of miR-498, as well as miR-146a-5p, resulted in reduced proliferation in comparison to negative controls. These results indicate that down-regulation of *BRCA1* by miR-498 could promote proliferation and contribute to tumorigenesis.

## DISCUSSION

In the present study we identified miRNAs related to breast cancer subtypes and demonstrated that miR-498, a triple negative-specific miRNA, regulates *BRCA1* expression. Because hundreds of miRNAs are currently predicted to target *BRCA1* 3′UTR (202 according to TargetScan database), but only 6 have been experimentally validated to date [10, 23-27], this finding adds important information about *BRCA1* regulation by miRNAs.

We first compared the miRNA expression profile of each of the main breast cancer subtypes (luminal A, luminal B, Her2 and triple negative) with the profile of healthy breast tissues, and identified subtype-specific miRNAs. Comparison of these results with the ones obtained by Blenkiron and colleages in the seminal study on miRNA expression profiling of breast cancer molecular subtypes [18] revealed similar patterns of expression for several key miRNAs (Supplementary Figure S1). Nevertheless, to our knowledge, this is the first report that takes into account healthy breast tissues to obtain miRNAs associated with breast cancer subtypes. We believe that this approach might be more appropriate since it allows for the identification of specifically deregulated miRNAs. Still, a limitation of this study is that our series of healthy breast tissues comes from women who have undergone breast reduction surgery, and are younger (mean age = 33 years) than the group of women with breast cancer (mean age = 60 years). Consequently we cannot reject an influence of age in the miRNA profile obtained, as it has been previously suggested [28, 29]. We could identify a great number of specifically deregulated miRNAs for triple negative tumors. Of note, several members of the let-7 family (let-7d-5p, let-7i-5p, let-7a-5p) were specifically underexpressed in our series of triple negative tumors. Let-7 is a family of miRNAs highly conserved across species and is often cited as the archetypal tumor-suppressing miRNA family. It has been shown that downregulation of let-7 promotes self-renewal and leads to a less differentiated cellular state in human and murine breast cells [30, 31]. Thus, misregulation of member of this family in triple negative breast tumors could explain at least in part why this subtype tends to grow and spread more quickly than other types of breast cancer and why triple negative cancer cells are often poorly differentiated.

The tumors used in this study were classified according to the combined evaluation of ER, PR, HER2 and Ki67 immunoreactivity. It is known that although the IHC subgroups approximate the molecular subtypes, there is not perfect overlap. As an example, some basal-like breast cancers do not show the expected triple negative immunophenotype, and not all the immunohistochemically TNBC are classified as basal-like by gene expression profiling [32]. Recently, a hormone receptor IHC cutpoint of <1% staining has been defined to more precisely classify the basal-like subgroup [33]. We believe that by using this cutpoint, our IHC classification is more accurate, although some discordance can still exist. This discordance could be the reason for the heterogeneity observed in the miRNA expression profile of our series of triple negative tumors (Figure 1B). Another reason for this heterogeneity could be the presence of *BRCA1* mutations. Women diagnosed with TNBC younger than 50 years have increased likelihood of carrying a *BRCA1* mutation [34]. In our series of triple negative patients, seven women were younger than 50 years when diagnosed for TNBC, and two out of them tested positive for BRCA1 mutations. Neverthelles, these two women did not show a different miRNA expression profile within the TNBC group. Therefore, other factors may be responsible for the differences observed in the miRNA expression profile.

We found that the identified triple negative-specific miRNAs were predicted to target several pathways related to cell proliferation and migration such as regulation of actin cytoskeleton, focal adhesion, PI3K-Akt, MAPK and Wnt signaling pathways. Interestingly, the most significantly enriched pathway, PI3K-Akt, is frequently activated in TNBC [35], and PI3K inhibitors are currently undergoing clinical investigation in patients with these cancers [36]. Thus, identification of deregulated pathways in TNBC is of great relevance since it can provide new therapeutic approaches for the treatment of this poor-prognosis subtype.

Several studies have shown that most sporadic triple negative tumors have a reduced expression of the *BRCA1* gene [7, 8], but mutations in this gene are rare. In this way, we investigated if any of the 78 miRNAs specifically overexpressed in triple negative breast tumors have binding sites in the 3′UTR of *BRCA1*, thus reducing *BRCA1* expression. MiR-498, miR-187-5p and miR-146a-5p were predicted with high scores to target the 3′UTR of *BRCA1*, and we functionally validated these results by luciferase reporter assay. MiRNA-146a-5p has previously been reported to bind to *BRCA1* 3′UTR and its expression in breast cancer cells has been associated with increased proliferation and reduced homologous recombination [10, 20]. The role of miR-498 in cancer development has not been well documented. While it seems to be downregulated in some cancers such as colon and ovarian cancers [37, 38], its overexpression has been reported in metastatic medullary thyroid carcinoma and retinoblastoma [39, 40]. Similarly, high levels of miR-187-5p have been associated with ovarian cancer [41] but its downregulation has been reported in clear cell renal cell carcinoma and prostate cancer [42, 43]. Interestingly, its overexpression has been associated with poor outcome in breast cancer, leading to a more aggressive phenotype [44].

Although we did not observed overexpression of miR-187-5p in the analyzed triple negative cell lines, it is noteworthy that we used a reduced number of cell lines and they might not represent what we found in the patients. The analysis of an extended set of breast cancer cell lines is recommended in order to better understand the relationship between miR-187-5p and *BRCA1* expression. On the other hand, we found that miR-498 was strongly expressed in the triple negative cell line Hs578T and its expression was negatively correlated with *BRCA1* expression levels. However, we did not observed an overexpression in the other triple negative cell line, MDA-MB-231. In this cell line, other mechanisms might be responsible for the reduced expression of *BRCA1*. In fact, it has been reported that MDA-MB-231 cells are hemizygous in *BRCA1* [45]. Therefore the loss of one allele in combination with other modifiers of gene expression could explain the low levels of *BRCA1* in this cell line.

We functionally demonstrated the negative correlation between miR-498 and *BRCA1* in breast cancer cell lines: inhibition of miR-498 in Hs578T cells increased *BRCA1* levels and its overexpression in MCF7 cells reduced *BRCA1* expression. These results suggest that miR-498 inhibits *BRCA1* expression in breast cancer. In addition, we have demonstrated that miR-498 inhibition leads to reduced proliferation of triple negative cells. Since induction of *BRCA1* expression has been shown to inhibit growth in breast tumors and cell lines [21], this result is consistent with a role of miR-498 in the inhibition of *BRCA1*. Hence, our data support that miR-498 promotes cell proliferation in triple negative breast cancer through direct regulation of *BRCA1* expression. These findings confirm previous studies that suggest that miRNA deregulation might be involved in the inactivation of *BRCA1* in sporadic breast cancer [10, 23-25].

In summary, we have identified miRNAs specific for the main subtypes of breast cancer and determined biological processes potentially deregulated in TNBC. In addition, this study sheds light on the mechanisms behind the decreased expression of *BRCA1* in sporadic TNBC by adding a new miRNA that regulates *BRCA1*. Determination of these mechanisms is essential to increase our understanding of TNBC etiology and to permit better therapeutic approaches.

## MATERIALS AND METHODS

All the selection and processing of tissues, as well as RNA extraction and microarray hybridization, have been already published [46], and will be briefly described.

### Breast tumors and healthy breast tissues

The formalin-fixed paraffin-embedded (FFPE) breast tissues used for microarray profiling consisted of 122 breast tumor samples obtained from patients undergoing surgery for breast cancer and 11 normal breast tissue samples obtained after breast reduction surgery from healthy women with no family history of cancer. Tissue blocks were obtained from two Spanish hospitals: Hospital Virgen de la Macarena (Sevilla) and Hospital Monte Naranco (Oviedo), and from one biobank: the INCLIVA BioBank (PT13/0010/0004) integrated in the Spanish National Biobanks Network. Informed consent was acquired from all patients and the study was approved by the ethics committee of Instituto de Salud Carlos III, Hospital Virgen de la Macarena, Hospital Monte Naranco and INCLIVA BioBank. All samples were histologically confirmed by two pathologists (RG-C, PM) and classified as triple negative (n=31), Her2 (n=27), luminal B (n=33) or luminal A (n=31) tumors based on the expression of inmunohistochemical markers (ER, PR, c-erb B2 and Ki-67), following the criteria adopted in the 13th St Gallen International Breast Cancer Conference [47]. In order to obtain a more accurate classification, an ER and PR cutpoint of <1% staining was used [33]. In the case of a weak positive reaction of c-erb B2, fluorescent in situ hybridization was performed to confirm the overexpression of this receptor. Clinicopathologic data are summarized in Supplementary Tables S2 and S3.

### Cell lines and cell culture

Five human cell lines representative of the main molecular subtypes of breast cancer, MDA-MB-231 (triple negative), Hs578T (triple negative), SKBR3 (Her2), BT474 (luminal B) and MCF7 (luminal A), were obtained from the Cancer Epigenetics Group at the Bellvitge Institute for Biomedical Research (Barcelona, Spain). In addition, a sixth cell line derived from an embryonic kidney, HEK-293T, was acquired. Cell lines were grown in RPMI-1640 (Sigma-Aldrich) or Dulbecco's Modified Eagle's Medium (DMEM, Sigma-Aldrich) containing 10% fetal bovine serum, 1% penicillin/streptomycin and 0.5% fungizone (Gibco, Life Technologies). In the case of BT474 cells, the medium was completed with 0.01 mg/ml of insulin (Sigma-Aldrich).

### MiRNA microarray hybridization and data analysis

Total RNA was extracted from macrodissected FFPE tissues using miRNeasy FFPE Kit (Qiagen), according to the manufacturer's instructions. RNA quality and quantity were assessed by NanoDrop Spectophotometer (NanoDrop technologies). Total RNA was hybridized on LNA-based miRNA microarrays (7th generation) containing probes for 1919 human miRNAs in quadruplicate, as described in [46]. Microarraybackground subtraction, data normalization, clustering and differential expression analysis was performed. Microarray dataset is publically available at NCBI's Gene Expression Omnibus database http://www.ncbi.nlm.nih.gov/geo/ under GEO accession number GSE58606.

### MiRNA target prediction algorithms

miRGate [19] has been used to find miRNAs targeting *BRCA1* 3′UTR. Briefly, miRGate is a freely available database that contains predicted as well as experimentally validated target pairs from five well-recognized algorithms calculated from a common and comprehensive dataset. It includes TargetScan (http://www.targetscan.org/), miRanda (http://www.microRNA.org/), MicroTar (http://tiger.dbs.nus.edu.sg/microtar/), Pita (http://genie.weizmann.ac.il/pubs/mir07/mir07_dyn_data.html), and RNAHybrid (http://bibiserv.techfak.uni-bielefeld.de/rnahybrid.

### Luciferase reporter assay

The 3′UTR of *BRCA1* was cloned in the pGL3-Control vector (Promega), downstream to firefly luciferase. Pre-miRNA oligonucleotides (pre-miR-498, pre-miR-187-5p, pre-miR-146a-5p and non-targeting control) were purchased from Exiqon and transfected together with the firefly luciferase-*BRCA1* 3′UTR construct and the renilla luciferase vector into 293T cells in a 96-well plate format using Lipofectamine 2000 reagent (Invitrogen). Cells were grown for 48h, after which luciferase activity was assayed with Dual-Glo Luciferase Assay System (Promega) according to manufacturer's instructions. Experiments were performed in triplicate and normalization was achieved using Renilla luciferase activity.

### Quantitative real time PCR (qRT-PCR)

Total RNA from cell lines was extracted using miRNeasy Mini Kit (Qiagen). Quantification of the expression of miR-498 and miR-187-5p was performed by qRT-PCR using miRCURY LNA™ Universal RT microRNA PCR system (Exiqon) according to the manufacturer's protocol, as previously described [17]. *BRCA1* expression was analyzed by qPCR using TaqMan assays. Five hundred nanograms of total RNA was reverse transcribed using the High-Capacity cDNA Reverse Transcription Kit (Applied Biosystems) and random primers following manufacturer's instructions. The cDNA was then 10x diluted and amplified by qPCR with the use of FAM/NFQ fluorescently labeled probes TaqMan (Roche Universal Probe library, Roche), specific primers (Sigma-Aldrich) and TaqMan Universal PCR Maser Mix (Applied Biosystems). Gene-specific primers were as follows: *BRCA1* F, ttaaagaaagaaaaatgctga, R, ggtggtttcttccattgacc (probe 82); *ACTB* F, ccaaccgcgagaagatga, R, ccagaggcgtacagggatag (probe 64); *MRLP19* F, ggaatgttatcgaaggacaag, R, caggaagggcatctcgtaag (probe 42). mRNA expression levels were detected using ABI Prism Sequence Detection System 7900HT (Applied Biosystems). All reactions were performed in triplicate and no-template controls were included in each run. Normalization of mRNA expression was carried out using *ACTB* and *MRLP19* as reference genes. Relative expression was calculated using the comparative cycle threshold (ΔΔCt) method implemented in qBasePLUS software (Biogazelle).

### MiR-498 expression and inhibition in breast cancer cell lines

Synthetic pre-miR-498, anti-miR-498 and non-targeting control were transfected into MCF7 and Hs578T cells using Oligofectamine reagent (Invitrogen), following the manufacturer's instructions. Pre-miRNA and anti-miRNA oligonucleotides were purchased from Exiqon. One day before transfection, cells were seeded in 6-well (for qRT-PCR) or 100 mm (for western blot) plates in antibiotic-free media to a density of 60%. At 48 hours after transfection cells were harvested for mRNA and/or protein analysis.

### Western blot analysis

Since BRCA1 protein is predominantly expressed in the nucleus, nuclear protein extraction was performed. Cells were lysed with RSB buffer (Tris 10 mM pH 7.5, NaCl 10 mM and MgCl2 3 mM,) containing protease inhibitor (Roche). Cell pellets were then dissolved in NB buffer (Tris 10 mM pH 7.5, NaCl 0.4 mM and EDTA 1 mM) containing protease inhibitor and supernatant was collected. Protein concentration was measured by Lowry assay method (Bio-Rad) and equal amounts of protein (50μg) were separated by SDS-PAGE on 6% home-made gels. Proteins were then electrotransferred to nitrocellulose membrane (Whatman), before blocking and incubation with antibodies. Antibodies used in this study were mouse anti-BRCA1 (OP92, Calbiochem) at 1:100 dilution, mouse anti-HSP70/HSC70 (ADI-SPA-820, Enzo Life Sciences) at 1:2000 dilution (loading control) and the corresponding horseradish peroxidase (HRP) conjugated secondary antibody (Dako, Glostrup, Denmark) at 1:10000 dilution. Antibody visualization was carried out with Amersham ECL Western Blotting Detection Reagent (GE Healthcare Life Sciences). BRCA1 protein content was determined relative to HSP70/HSC70 protein content.

### Cell proliferation assay

Cell proliferation assay was assessed by using water-soluble tetrazolium salt (WST-1) assay. Hs578T cells were seeded in 24-well plates one day before transfection at 30-40% confluence in antibiotic-free media. Cells were transfected using Oligofectamine (Invitrogen) with 50 nM of anti-miR-498, anti-miR-146a-5p, non-targeting miRNA inhibitor or mock transfection control. Cell viability was determined at 24, 48 and 72 hours after transfection. Cells were incubated in 25μl of WST-1 (Roche) diluted in 500 μl normal culture medium at 37°C for 1h. Each value represents the average of four independent replicates.

## SUPPLEMENTARY FIGURE AND TABLES



## References

[1] Perou CM, Sorlie T, Eisen MB, van de Rijn M, Jeffrey SS, Rees CA, Pollack JR, Ross DT, Johnsen H, Akslen LA, Fluge O, Pergamenschikov A, Williams C, Zhu SX, Lonning PE, Borresen-Dale AL (2000). Molecular portraits of human breast tumours. Nature.

[2] Sorlie T, Perou CM, Tibshirani R, Aas T, Geisler S, Johnsen H, Hastie T, Eisen MB, van de Rijn M, Jeffrey SS, Thorsen T, Quist H, Matese JC, Brown PO, Botstein D, Lonning PE (2001). Gene expression patterns of breast carcinomas distinguish tumor subclasses with clinical implications. Proceedings of the National Academy of Sciences of the United States of America.

[3] Dent R, Trudeau M, Pritchard KI, Hanna WM, Kahn HK, Sawka CA, Lickley LA, Rawlinson E, Sun P, Narod SA (2007). Triple-negative breast cancer: clinical features and patterns of recurrence. Clinical cancer research.

[4] Narod SA, Foulkes WD (2004). BRCA1 and BRCA2: 1994 and beyond. Nature reviews Cancer.

[5] Drost R, Jonkers J (2014). Opportunities and hurdles in the treatment of BRCA1-related breast cancer. Oncogene.

[6] Turner NC, Reis-Filho JS (2006). Basal-like breast cancer and the BRCA1 phenotype. Oncogene.

[7] Turner NC, Reis-Filho JS, Russell AM, Springall RJ, Ryder K, Steele D, Savage K, Gillett CE, Schmitt FC, Ashworth A, Tutt AN (2007). BRCA1 dysfunction in sporadic basal-like breast cancer. Oncogene.

[8] Mueller CR, Roskelley CD (2003). Regulation of BRCA1 expression and its relationship to sporadic breast cancer. Breast cancer research: BCR.

[9] Wilson CA, Ramos L, Villasenor MR, Anders KH, Press MF, Clarke K, Karlan B, Chen JJ, Scully R, Livingston D, Zuch RH, Kanter MH, Cohen S, Calzone FJ, Slamon DJ (1999). Localization of human BRCA1 and its loss in high-grade, non-inherited breast carcinomas. Nature genetics.

[10] Garcia AI, Buisson M, Bertrand P, Rimokh R, Rouleau E, Lopez BS, Lidereau R, Mikaelian I, Mazoyer S (2011). Down-regulation of BRCA1 expression by miR-146a and miR-146b-5p in triple negative sporadic breast cancers. EMBO molecular medicine.

[11] Iorio MV, Ferracin M, Liu CG, Veronese A, Spizzo R, Sabbioni S, Magri E, Pedriali M, Fabbri M, Campiglio M, Menard S, Palazzo JP, Rosenberg A, Musiani P, Volinia S, Nenci I (2005). MicroRNA gene expression deregulation in human breast cancer. Cancer research.

[12] Farazi TA, Horlings HM, Ten Hoeve JJ, Mihailovic A, Halfwerk H, Morozov P, Brown M, Hafner M, Reyal F, van Kouwenhove M, Kreike B, Sie D, Hovestadt V, Wessels LF, van de Vijver MJ, Tuschl T (2011). MicroRNA sequence and expression analysis in breast tumors by deep sequencing. Cancer research.

[13] Persson H, Kvist A, Rego N, Staaf J, Vallon-Christersson J, Luts L, Loman N, Jonsson G, Naya H, Hoglund M, Borg A, Rovira C (2011). Identification of new microRNAs in paired normal and tumor breast tissue suggests a dual role for the ERBB2/Her2 gene. Cancer research.

[14] Sempere LF, Christensen M, Silahtaroglu A, Bak M, Heath CV, Schwartz G, Wells W, Kauppinen S, Cole CN (2007). Altered MicroRNA expression confined to specific epithelial cell subpopulations in breast cancer. Cancer research.

[15] Volinia S, Calin GA, Liu CG, Ambs S, Cimmino A, Petrocca F, Visone R, Iorio M, Roldo C, Ferracin M, Prueitt RL, Yanaihara N, Lanza G, Scarpa A, Vecchione A, Negrini M (2006). A microRNA expression signature of human solid tumors defines cancer gene targets. Proceedings of the National Academy of Sciences of the United States of America.

[16] Volinia S, Galasso M, Sana ME, Wise TF, Palatini J, Huebner K, Croce CM (2012). Breast cancer signatures for invasiveness and prognosis defined by deep sequencing of microRNA. Proceedings of the National Academy of Sciences of the United States of America.

[17] Matamala N, Vargas MT, Gonzalez-Campora R, Minambres R, Arias JI, Menendez P, Andres-Leon E, Gomez-Lopez G, Yanowsky K, Calvete-Candenas J, Inglada-Perez L, Martinez-Delgado B, Benitez J (2015). Tumor MicroRNA Expression Profiling Identifies Circulating MicroRNAs for Early Breast Cancer Detection. Clinical chemistry.

[18] Blenkiron C, Goldstein LD, Thorne NP, Spiteri I, Chin SF, Dunning MJ, Barbosa-Morais NL, Teschendorff AE, Green AR, Ellis IO, Tavare S, Caldas C, Miska EA (2007). MicroRNA expression profiling of human breast cancer identifies new markers of tumor subtype. Genome biology.

[19] Andres-Leon E, Gonzalez Pena D, Gomez-Lopez G, Pisano DG (2015). miRGate: a curated database of human, mouse and rat miRNA-mRNA targets. Database: the journal of biological databases and curation.

[20] Shen J, Ambrosone CB, DiCioccio RA, Odunsi K, Lele SB, Zhao H (2008). A functional polymorphism in the miR-146a gene and age of familial breast/ovarian cancer diagnosis. Carcinogenesis.

[21] Holt JT, Thompson ME, Szabo C, Robinson-Benion C, Arteaga CL, King MC, Jensen RA (1996). Growth retardation and tumour inhibition by BRCA1. Nature genetics.

[22] Thompson ME, Jensen RA, Obermiller PS, Page DL, Holt JT (1995). Decreased expression of BRCA1 accelerates growth and is often present during sporadic breast cancer progression. Nature genetics.

[23] Tan X, Peng J, Fu Y, An S, Rezaei K, Tabbara S, Teal CB, Man YG, Brem RF, Fu SW (2014). miR-638 mediated regulation of BRCA1 affects DNA repair and sensitivity to UV and cisplatin in triple-negative breast cancer. Breast cancer research: BCR.

[24] He X, Xiao X, Dong L, Wan N, Zhou Z, Deng H, Zhang X (2014). MiR-218 regulates cisplatin chemosensitivity in breast cancer by targeting BRCA1. Tumour biology.

[25] Moskwa P, Buffa FM, Pan Y, Panchakshari R, Gottipati P, Muschel RJ, Beech J, Kulshrestha R, Abdelmohsen K, Weinstock DM, Gorospe M, Harris AL, Helleday T, Chowdhury D (2011). miR-182-mediated downregulation of BRCA1 impacts DNA repair and sensitivity to PARP inhibitors. Molecular cell.

[26] Sun C, Li N, Yang Z, Zhou B, He Y, Weng D, Fang Y, Wu P, Chen P, Yang X, Ma D, Zhou J, Chen G (2013). miR-9 regulation of BRCA1 and ovarian cancer sensitivity to cisplatin and PARP inhibition. Journal of the National Cancer Institute.

[27] Shen J, Ambrosone CB, Zhao H (2009). Novel genetic variants in microRNA genes and familial breast cancer. International journal of cancer.

[28] ElSharawy A, Keller A, Flachsbart F, Wendschlag A, Jacobs G, Kefer N, Brefort T, Leidinger P, Backes C, Meese E, Schreiber S, Rosenstiel P, Franke A, Nebel A (2012). Genome-wide miRNA signatures of human longevity. Aging cell.

[29] Noren Hooten N, Abdelmohsen K, Gorospe M, Ejiogu N, Zonderman AB, Evans MK (2010). microRNA expression patterns reveal differential expression of target genes with age. PloS one.

[30] Ibarra I, Erlich Y, Muthuswamy SK, Sachidanandam R, Hannon GJ (2007). A role for microRNAs in maintenance of mouse mammary epithelial progenitor cells. Genes & development.

[31] Yu F, Yao H, Zhu P, Zhang X, Pan Q, Gong C, Huang Y, Hu X, Su F, Lieberman J, Song E (2007). let-7 regulates self renewal and tumorigenicity of breast cancer cells. Cell.

[32] Carey L, Winer E, Viale G, Cameron D, Gianni L (2010). Triple-negative breast cancer: disease entity or title of convenience?. Nature reviews Clinical oncology.

[33] Cheang MC, Martin M, Nielsen TO, Prat A, Voduc D, Rodriguez-Lescure A, Ruiz A, Chia S, Shepherd L, Ruiz-Borrego M, Calvo L, Alba E, Carrasco E, Caballero R, Tu D, Pritchard KI (2015). Defining breast cancer intrinsic subtypes by quantitative receptor expression. The oncologist.

[34] Robertson L, Hanson H, Seal S, Warren-Perry M, Hughes D, Howell I, Turnbull C, Houlston R, Shanley S, Butler S, Evans DG, Ross G, Eccles D, Tutt A, Rahman N, TMG TNTT (2012). BRCA1 testing should be offered to individuals with triple-negative breast cancer diagnosed below 50 years. British journal of cancer.

[35] Cancer Genome Atlas N (2012). Comprehensive molecular portraits of human breast tumours. Nature.

[36] Gordon V, Banerji S (2013). Molecular pathways: PI3K pathway targets in triple-negative breast cancers. Clinical cancer research.

[37] Gopalan V, Smith RA, Lam AK (2015). Downregulation of microRNA-498 in colorectal cancers and its cellular effects. Exp Cell Res.

[38] Kasiappan R, Shen Z, Tse AK, Jinwal U, Tang J, Lungchukiet P, Sun Y, Kruk P, Nicosia SV, Zhang X, Bai W (2012). 1,25-Dihydroxyvitamin D3 suppresses telomerase expression and human cancer growth through microRNA-498. J Biol Chem.

[39] Santarpia L, Calin GA, Adam L, Ye L, Fusco A, Giunti S, Thaller C, Paladini L, Zhang X, Jimenez C, Trimarchi F, El-Naggar AK, Gagel RF (2013). A miRNA signature associated with human metastatic medullary thyroid carcinoma. Endocr Relat Cancer.

[40] Zhao JJ, Yang J, Lin J, Yao N, Zhu Y, Zheng J, Xu J, Cheng JQ, Lin JY, Ma X (2009). Identification of miRNAs associated with tumorigenesis of retinoblastoma by miRNA microarray analysis. Childs Nerv Syst.

[41] Chao A, Lin CY, Lee YS, Tsai CL, Wei PC, Hsueh S, Wu TI, Tsai CN, Wang CJ, Chao AS, Wang TH, Lai CH (2012). Regulation of ovarian cancer progression by microRNA-187 through targeting Disabled homolog-2. Oncogene.

[42] Zhao J, Lei T, Xu C, Li H, Ma W, Yang Y, Fan S, Liu Y (2013). MicroRNA-187, down-regulated in clear cell renal cell carcinoma and associated with lower survival, inhibits cell growth and migration though targeting B7-H3. Biochem Biophys Res Commun.

[43] Fuse M, Kojima S, Enokida H, Chiyomaru T, Yoshino H, Nohata N, Kinoshita T, Sakamoto S, Naya Y, Nakagawa M, Ichikawa T, Seki N (2012). Tumor suppressive microRNAs (miR-222 and miR-31) regulate molecular pathways based on microRNA expression signature in prostate cancer. J Hum Genet.

[44] Mulrane L, Madden SF, Brennan DJ, Gremel G, McGee SF, McNally S, Martin F, Crown JP, Jirstrom K, Higgins DG, Gallagher WM, O'Connor DP (2012). miR-187 is an independent prognostic factor in breast cancer and confers increased invasive potential in vitro. Clin Cancer Res.

[45] Drew Y, Mulligan EA, Vong WT, Thomas HD, Kahn S, Kyle S, Mukhopadhyay A, Los G, Hostomsky Z, Plummer ER, Edmondson RJ, Curtin NJ (2011). Therapeutic potential of poly(ADP-ribose) polymerase inhibitor AG014699 in human cancers with mutated or methylated BRCA1 or BRCA2. Journal of the National Cancer Institute.

[46] Matamala N, Vargas MT, Gonzalez-Campora R, Minambres R, Arias JI, Menendez P, Andres-Leon E, Gomez-Lopez G, Yanowsky K, Calvete-Candenas J, Inglada-Perez L, Martinez-Delgado B, Benitez J (2015). Tumor microRNA expression profiling identifies circulating microRNAs for early breast cancer detection. Clinical chemistry.

[47] Goldhirsch A, Winer EP, Coates AS, Gelber RD, Piccart-Gebhart M, Thurlimann B, Senn HJ, Panel m (2013). Personalizing the treatment of women with early breast cancer: highlights of the St Gallen International Expert Consensus on the Primary Therapy of Early Breast Cancer 2013. Annals of oncology.

