# Supplementary material for: MicroRNA deregulation in triple negative breast cancer reveals a role of miR-498 in regulating BRCA1 expression
Source: Oncotarget. 2016 Feb 25;7(15):20068–79. doi: 10.18632/oncotarget.7705 (PMC4991439; doi:10.18632/oncotarget.7705)
Supplement: Supplementary file 1 [file oncotarget-07-20068-s001.pdf]

# SUPPLEMENTARY FIGURE AND TABLES

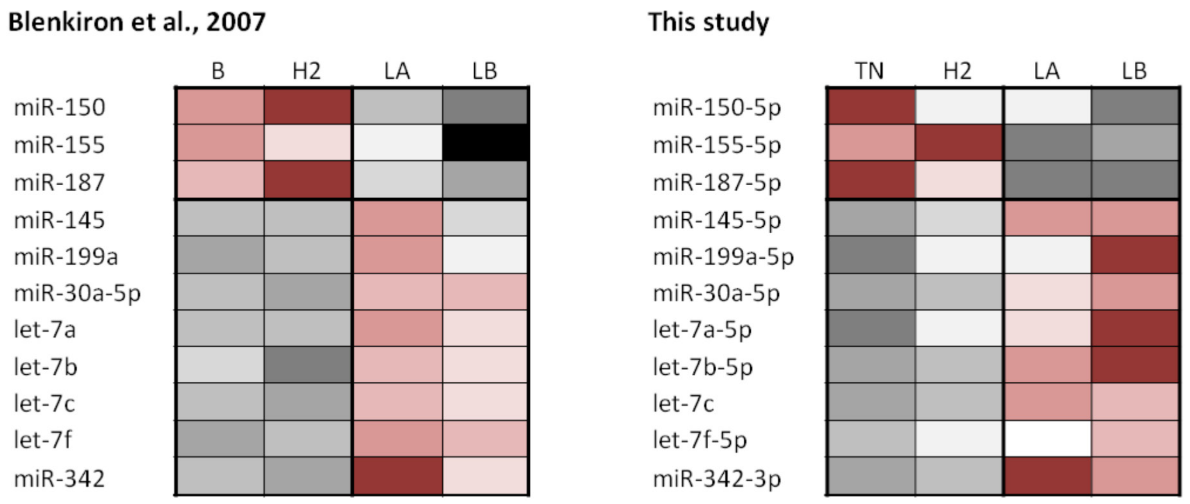


**Supplemental Figure S1: Subtype-associated miRNAs identified in the seminal study by Blenkiron et al., with a similar pattern of expression in the present study.** Colors represent the average expression value for each group of interest: dark red indicates high expression and dark grey, low expression.

**Supplementary Table S1: Specifically up or downregulated miRNAs in breast cancer molecular subtypes after comparison with normal breast tissues**

| Molecular subtype | Deregulation | Number | miRNAs |
|---|---|---|---|
| **Triple negative** | Up | 78 | miR-642b-5p,miR-4795-3p,miR-4653-3p,miR-3124-3p,miR-4501,miR-4698,miR-1973,miR-3976,miR-4417,miR-634,miR-4723-5p,miR-150-5p,miR-4639-3p,miR-659-5p,miR-4633-5p,miR-5584-3p,miR-4449,miR-921,miR-5000-3p,miR-4707-3p,miR-513a-5p,miR-519e-5p,miR-552,miR-4431,miR-4677-3p,miR-4329,miR-1273e,miR-711,miR-4503,miR-3687,miR-492,miR-3591-5p,miR-5588-3p,miR-874,miR-181a-2-3p,miR-4636,miR-498,miR-4782-5p,miR-490-5p,miR-548ao-3p,miR-5193,miR-3648,miR-4511,miR-1827,miR-187-5p,miR-550b-2-5p,miR-4674,miR-3912,let-7d-3p,miR-2113,miR-4264,miR-675-3p,miR-5689,miR-4784,miR-1285-5p,miR-4694-5p,miR-5089,miR-5187-5p,miR-650,miR-506-5p,miR-891a,miR-1265,miR-1197,miR-3944-5p,miR-4683,miR-4536-3p,miR-3618,miR-4535,miR-4778-5p,miR-3925-5p,miR-1321,miR-4436b-5p,miR-3161,miR-5006-3p,miR-3606,miR-146a-5p,miR-5580-5p |
| | Down | 27 | miR-554,miR-4444,miR-629-5p,miR-10a-5p,miR-140-3p,miR-374a-5p,miR-124-5p,let-7d-5p,miR-4791,miR-548o-3p,miR-1202,miR-4328,miR-448,miR-382-3p,miR-29b-2-5p,miR-590-3p,miR-10b-5p,let-7i-5p,miR-4301,miR-126-3p,miR-199a-5p,miR-3607-3p,miR-214-3p,miR-491-3p,miR-4285,miR-5701,let-7a-5p |
| **Her2** | Up | 0 | - |
| | Down | 1 | miR-574-3p |
| **Luminal B** | Up | 23 | miR-16-5p,let-7g-5p,miR-23a-3p,miR-200c-3p,miR-34a-5p,miR-101-3p,miR-26b-5p,miR-193a-3p,miR-30b-5p,miR-20a-5p,miR-29a-3p,miR-15a-5p,miR-27b-3p,miR-93-5p,miR-4714-5p,miR-1280,miR-29b-3p,miR-15b-5p,miR-26a-5p,miR-374b-5p,miR-429,miR-107 |
| | Down | 16 | miR-184,miR-4462,miR-4649-5p,miR-1299,miR-4433-3p,miR-4472,miR-302a-3p,miR-4507,miR-3646,miR-371b-5p,miR-4497,miR-4787-5p,miR-548ap-5p,miR-548j,miR-3940-5p,miR-4505,miR-1275 |
| **Luminal A** | Up | 5 | miR-4421,miR-3667-5p,miR-5196-3p,miR-548k,miR-331-3p |
| | Down | 12 | miR-486-5p,miR-4492,miR-664-3p,miR-124-3p,miR-4446-5p,miR-3664-5p,miR-513b,miR-3620,miR-4769-3p,miR-4646-3p,miR-3182,miR-4723-3p |
| **Common** | Up | 25 | miR-21-5p,miR-3613-3p,miR-4668-5p,miR-106b-5p,miR-200a-3p,miR-5704,miR-1278,miR-182-5p,miR-1264,miR-96-5p,miR-190b,miR-3611,miR-185-5p,miR-339-5p,miR-7-5p,miR-340-5p,miR-203,miR-342-5p,miR-1244,miR-600,miR-760,miR-92a-2-5p,miR-219-2-3p,miR-553,miR-4521 |
| | Down | 27 | miR-889,miR-192-3p,miR-432-5p,miR-1224-5p,miR-1205,miR-1228-5p,miR-571,miR-1229,miR-4253,miR-2276,miR-577,miR-548s,miR-744-3p,miR-1207-5p,miR-1270,miR-877-5p,miR-499a-5p,miR-574-5p,miR-718,miR-4695-3p,miR-3164,miR-3148,miR-3941,miR-4488,miR-4516,miR-3656,miR-125b-5p |

**Supplementary Table S2: Clinicopathologic data of breast cancer patients at the time of diagnosis**

| Characteristics | Breast cancer patients (n=122) |
|---|---|
| **Age** | n=89 (27% unknown) |
| Mean, years | 60 |
| Range, years | 28-89 |
| <35y | 5 (6%) |
| 35-45y | 7 (8%) |
| 45-55y | 27 (30%) |
| 55-65y | 15 (17%) |
| 65-75y | 14 (16%) |
| >75y | 21 (23%) |
| **Tumor size** | n=78 (36% unknown) |
| T1 (≤ 2cm) | 44 (56%) |
| T2 (2.1-5cm) | 28 (36%) |
| T3 o T4 (>5cm) | 6 (8%) |
| **Grade** | n=77 (37% unknown) |
| I | 5 (7%) |
| II | 25 (32%) |
| III | 47 (61%) |
| **Nodes** | n=78 (36% unknown) |
| negative | 29 (37%) |
| positive | 49 (63%) |
| **Stage** | n=78 (36% unknown) |
| I | 24 (31%) |
| II | 29 (37%) |
| III | 17 (22%) |
| IV | 8 (10%) |
| **ER** | n=122 (0% unknown) |
| negative | 58 (48%) |
| positive | 64 (52%) |
| **PR** | n=120 (2% unknown) |
| negative | 60 (50%) |
| positive | 60 (50%) |
| **KI-67** | n=118 (3% unknown) |
| low (<14%) | 32 (27%) |
| intermediate-high (≥14%) | 86 (73%) |
| **Her2** | n=121 (1% unknown) |

(*Continued*)

| Characteristics | Breast cancer patients (n=122) |
|---|---|
| negative | 90 (74%) |
| positive | 31 (26%) |
| **Molecular subtype** | n=122 (0% unknown) |
| LA | 31 (25%) |
| LB | 33 (27%) |
| H2 | 27 (23%) |
| TN | 31 (25%) |

**Supplementary Table S3: Age of breast cancer patients at the time of diagnosis according to molecular subtypes**

| | Triple negative | Her2 | Luminal B | Luminal A |
|---|---|---|---|---|
| Known cases | n=26 (16% unknown) | n=23 (15% unknown) | n=20 (40% unknown) | n=20 (36% unknown) |
| | 63 | 54 | 59 | 66 |
| Range, years | 34-88 | 28-85 | 30-85 | 40-89 |
| <35y | 1 (4%) | 2 (9%) | 2 (10%) | 0 (0%) |
| 35-45y | 1 (4%) | 3 (13%) | 2 (10%) | 1 (5%) |
| 45-55y | 9 (34%) | 7 (30%) | 5 (25%) | 6 (30%) |
| 55-65y | 2 (8%) | 7 (30%) | 4 (20%) | 1 (5%) |
| 65-75y | 5 (19%) | 2 (9%) | 2 (10%) | 6 (30%) |
| >75y | 8 (31%) | 2 (9%) | 5 (25%) | 6 (30%) |
